# The Contribution of Additional Sampling in Cholecystectomy Materials: A Multicenter Prospective Study

**DOI:** 10.5146/tjpath.2020.01483

**Published:** 2020-09-15

**Authors:** Samir Abdullazade, Fahire Göknur Akarca, Güldal Esendağlı, Nesrin Turhan, Esra Erden, Berna Savaş, Fatma Markoç, Deniz Tunçel, Banu Özgüven Yılmaz, Burcu Saka, Sevinç Hallaç Keser, Selma Şengiz Erhan, Zühal Gücin, Özgül Sağol, Anıl Aysal Ağalar, Sevinç Çelik, Hatice Özer, İpek Erbarut Seven, Çiğdem Ataizi Çelikel, Özgür Ekinci, Hatice Reyhan Eğilmez, Serdar Balcı, Gülen Akyol

**Affiliations:** Department of Pathology, İzmir Tepecik Education and Research Hospital, İzmir, Turkey; Department of Medical Pathology, Gazi University, Faculty of Medicine, Ankara, Turkey; Department of Pathology, Turkey Yüksek İhtisas Education and Research Hospital, Ankara, Turkey; Department of Medical Pathology, Ankara University, Faculty of Medicine, Ankara, Turkey; Department of Pathology, Dr. Abdurrahman Yurtaslan Ankara Oncology Education and Research Hospital, Ankara, Turkey; Şişli Etfal Education and Research Hospital, İstanbul, Turkey; Department of Medical Pathology, Medipol University, Faculty of Medicine, İstanbul, Turkey; Department of Pathology, Lütfi Kırdar Education and Research Hospital, İstanbul, Turkey; Okmeydanı Education and Research Hospital, İstanbul, Turkey; Department of Medical Pathology, Bezmialem Vakıf University, Faculty of Medicine, İstanbul, Turkey; Dokuz Eylül University, Faculty of Medicine, İzmir, Turkey; Bozok University, Faculty of Medicine, Yozgat, Turkey; Cumhuriyet University, Faculty of Medicine, Sivas, Turkey; Marmara University, Faculty of Medicine, İstanbul, Turkey; Pathologist, Ankara, Turkey

**Keywords:** Gallbladder, Cholecystectomy, Dysplasia, Cancer, Macroscopy

## Abstract

***Objective:*** Cholecystectomy materials are frequently encountered in routine practice. The aim of this study was to determine the true frequency of gallbladder lesions, the diagnostic consistency, and standardization of reports after macroscopic sampling and microscopic evaluation based on previously defined criteria.

***Material and Method:*** 14 institutions participated in the study within the Hepato-Pancreato-Biliary Pathology Study Group. Routinely examined cholecystectomies within the last year were included in the study in these institutions. Additional sampling was performed according to the indications and criteria. The number of blocks and samples taken in the first macroscopic examination and the number of blocks and samples taken in the additional sampling were determined and the rate of diagnostic contribution of the additional examination was determined.

***Results:*** A total of 5,244 cholecystectomy materials from 14 institutions were included in the study. Additional sampling was found to be necessary in 576 cases (10.98%) from all institutions. In the first macroscopic sampling, the mean of the numbers of samples was approximately 4 and the number of blocks was 2. The mean of the numbers of additional samples and blocks was approximately 8 and 4, respectively. The diagnosis was changed in 144 of the 576 new sampled cases while the remaining 432 stayed unaltered.

***Conclusion:*** In this study, it was observed that new sampling after the first microscopic examination of cholecystectomy materials contributed to the diagnosis. It was also shown that the necessity of having standard criteria for macroscopic and microscopic examination plays an important role in making the correct diagnosis.

## INTRODUCTION

Cholecystectomies are frequently encountered in the pathologists’ daily routine and are usually performed for various benign etiologies. The common opinion is that all cholecystectomy materials should undergo pathological examination (1). Furthermore, there are a few studies discussing the pathological examination of the entire cholecystectomy material (2). Gallbladder carcinomas are difficult to detect clinically and radiologically in the early stages and 75% of malignant cases are not resectable at the time of diagnosis (3). There are various methods for the macroscopic examination of cholecystectomy materials (4–8) and all these methods are important in detecting incidental gallbladder cancer. The Hepato-Pancreato-Biliary (HPB) Pathology Study Group has also conducted a multicenter retrospective study to assess gallbladder lesions and establish common macroscopy and microscopy protocols in our country (9).

There are still some problems in the microscopic approach to the epithelial lesions of the gallbladder. The lesions to be reported, and the indications and criteria for additional sampling are topics of discussion. Although cholecystectomy material is frequently encountered in routine pathology practice, gallbladder epithelial anomalies and neoplasms are uncommon (10,11). Gallbladder epithelial lesions include metaplastic lesions (antral/pyloric metaplasia, intestinal metaplasia), benign epithelial neoplasms (adenomas/adenomyomas), biliary intraepithelial neoplasia (BilIN) (dysplasia/carcinoma in situ), and invasive carcinomas (11). In the differential diagnosis of these lesions, especially the ones that are not macroscopically evident, there are inconsistencies among the observers in approaching these lesions in addition to the problems experienced due to the nature of the lesions.

Protocols that should be followed in the sampling and microscopic evaluation of the gallbladder remain important and controversial since high-grade dysplasia and even invasive carcinomas cannot be diagnosed macroscopically in general (12).

## MATERIALS and METHOD

**Figure 1:**
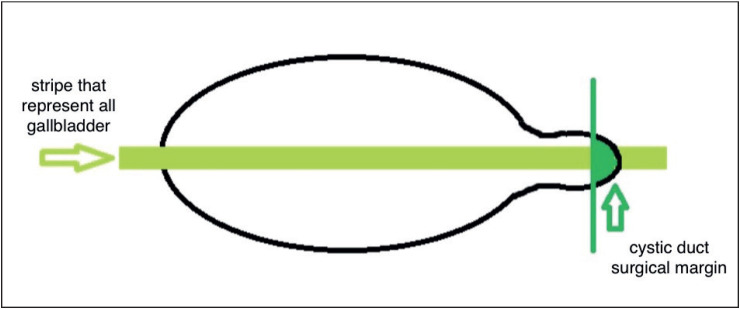
Macroscopic sampling method in gallbladder material.

A total of 23 pathologists from 14 institutions (8 University Hospitals and 6 Training and Research Hospitals) participated in this prospective study within the HPB Pathology Study Group. There is no expert consultation by a single qualified expert for dysplastic lesions. This study included cholecystectomy materials routinely analyzed in these institutions within the last year. We used a form for patient consent. In the first macroscopic examination of the material in accordance with the decided method, the surgical margin of the ductus cysticus was sampled in a way that the sectional side could be seen completely and was sampled completely by removing a full slice from the fundus to the ductus cysticus (Figure 1) (9). All polyps, if present, in the material (including cholesterol polyps) were sampled. Microscopic examinations were continued by examining the new samples in order to find out if any neoplastic polyp with focal epithelial atypia (including denuded epithelium), intestinal metaplasia, or high- or low-grade dysplasia were detected. In this method, the criteria defined for the reanalysis of macroscopic examination and for additional sampling (AS) were as follows (12):

1. If pyloric-gland metaplasia or mucinous pyloric gland nodules smaller than 3 mm is detected, there is no need for AS.
2. If a pyloric gland lesion larger than 3 mm is detected, the material and the container are reevaluated in terms of preinvasive papillary lesions. (No need for AS if the pathology is not observed.)
3. If intestinal metaplasia is detected, 2 cassettes of AS are taken as 2 to 3 samples per cassette.
4. If focal epithelial atypia is detected, 2 cassettes of AS are taken in the same way.
5. If severe atypia is found in addition to the denuded epithelium, 4 cassettes are taken in a similar way.
6. If high-grade dysplasia is detected, at least 12 cassettes are taken in the same way.
7. If a neoplastic polyp with dysplasia of any size is detected, the lesion is sampled completely and, in addition, five cassettes are obtained from the surrounding mucosa.
8. If invasive carcinoma is detected, 7-12 cassettes are taken to show the depth of the lesion and the relationship with the hepatic bed.

In accordance with these methods, the number of blocks and samples taken in the first macroscopic examination of the materials and after AS, as well as the number of new sampled cholecystectomy materials (cases), were reported. The rate of change in histopathological diagnosis following additional examinations of new sampled cholecystectomy materials was reported.

## RESULTS

**Figure 2:**
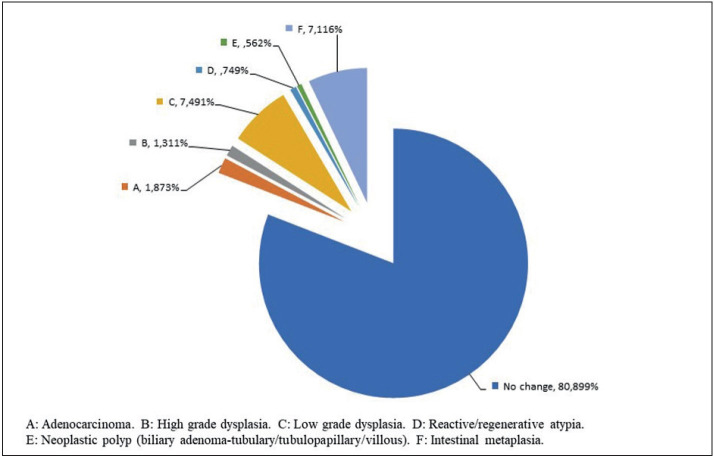
Frequency of bile duct lesions (all centers).

**Figure 3:**
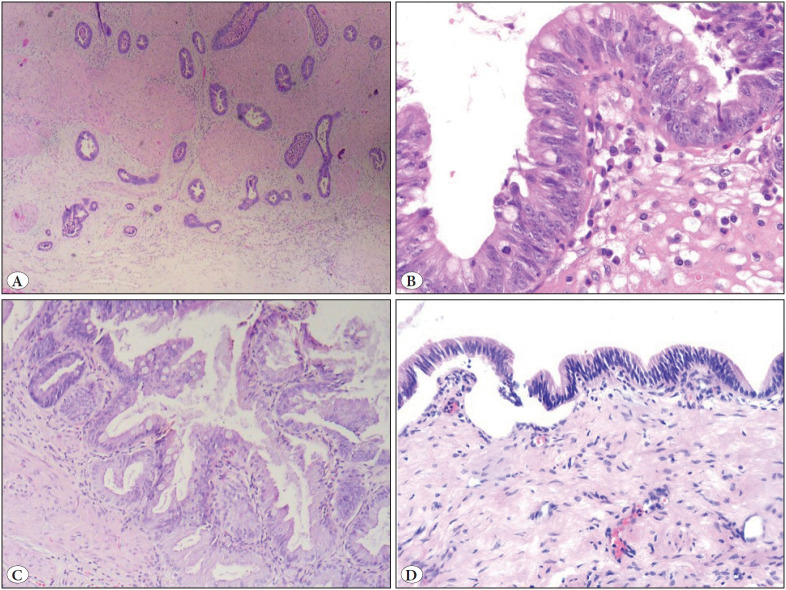
**A)** Adenocarcinoma (H&E; x40). **B)** High grade dysplasia (H&E; x200). **C)** Intestinal metaplasia (H&E; x100). **D)** Low grade dysplasia (H&E; x100).

A total of 5,244 cholecystectomy materials from 14 institutions were included in the study. AS was found to be necessary in 576 cases (10.98%) from all institutions. Males made up 189 patients while the remaining 387 were female. The average age was 54.3 years. The number of specimens in the first macroscopic sample ranged from 2 to 28 (mean 4.34) and the number of blocks ranged from 1 to 28 (mean 2.01). Among additional sampled cases, the number of additional samples varied between 2 and 51 (mean 8.04) and the number of additional blocks between 1 and 29 (mean 4). Of the 576 new sampled cases, 432 had no change in the diagnosis, while the diagnosis was changed in 144 cases. Adenocarcinoma was found in 10 cases, high-grade dysplasia in 7, low-grade dysplasia in 40, reactive/regenerative atypia in 4, neoplastic polyp in 3 (biliary adenoma-tubular, tubulopapillary, villous), and intestinal metaplasia in 38 (Figure 2, Figure 3).

## DISCUSSION

Epithelial lesions of gallbladder are common in routine practice since cholecystectomy is a frequently performed surgery (10,13). However, neoplasms and epithelial anomalies of the gallbladder are rare. Since even high-grade dysplasia and invasive carcinomas cannot be macroscopically detected in general (12), it is important to develop an easy-to-use, effective and up-to-date protocol for sampling as well as for microscopic evaluation of the gallbladder.

It has been reported that the clinical course is excellent in high-grade dysplasia cases in which invasive carcinoma was detected after AS and also in the in situ carcinoma/high-grade dysplasia cases in which the invasion was excluded after examining the entire material (14,15). In terms of treatment, one of the most important points to keep in mind is that cholecystectomy alone is sufficient in Tis and T1 tumors (16,17), whereas the surgical resection should be extended in cases where deeper invasion is detected (18,19).

Among other pathologies of the gallbladder, pyloric metaplasia is the most common type. Instead of this term, pseudopyloric, antral or mucosal gland hyperplasia is also used. Polyps with pyloric metaplasia, if larger than 1 cm, are categorized as neoplastic (or adenoma) in the presence of dysplasia in the metaplastic area. When chief and parietal cells are seen, heterotopia is the most likely diagnosis instead of metaplasia (10). Intestinal metaplasia, which is characterized by the presence of goblet cells, is less commonly seen than pyloric metaplasia.

It is however known that intestinal metaplasia is related to carcinoma, in a similar way as in the stomach (10). Examples of other metaplasia types include squamous, neuroendocrine, and pancreatic acinar cell metaplasia (10). AS is strongly suggested in cases where intestinal metaplasia is detected due to the carcinoma relationship described above. Considering the continuity of the gallbladder with bile ducts extending into the liver, the importance of pathology in the cholecystectomy material can be understood. Therefore, identification of all lesions (including hyperplasia and metaplasia) in cholecystectomy material in pathology reports is important both in terms of the database and clinical follow-up of the patients.

In our study, the rate of adenocarcinoma was 1.7% and this rate is compatible with the literature. The rate of incidental carcinoma in the literature ranges from 0.25% to 2% (20–22). In the case of adenocarcinoma found in a cholecystectomy material, the treatment plan also changes. Extension of the surgical resection area, lymphadenectomy, or chemo-radiotherapy can be applied to these patients. In this respect, it is important that the parameters required for treatment planning are included in the pathology report. The required parameters can be listed as the type of the procedure, histological type of the tumor, the location and size of the tumor, whether lymph nodes contain metastases or not, and the depth of invasion. In this regard, the macroscopy guide of the HPB Pathology Study Group including gallbladder carcinomas, has been updated and published on the website (23). The latest protocol, also recommended by the College of American Pathologists, emphasizes that the pathology report should include the localization, size, grade, spread, surgical margins of the tumor as well as lympho-vascular and perineural invasion, regional lymph nodes, grade, and additional histopathological findings such as dysplasia/adenoma, cholelithiasis, chronic and acute cholecystitis, intestinal metaplasia, diffuse calcification (porcelain gallbladder), and primary sclerosing cholangitis in the liver bed (24).

There are various methods in the literature for macroscopic examination and sampling of gallbladder resection materials (4–8). The issue of how many new samples should be taken in a case of dysplasia is still controversial (25,26). In addition, the criteria for discrimination between low-grade dysplasia and reactive atypia as well as true diagnosis of such lesions have not been clearly identified (27). Although the rate of dysplasia was reported to be 3.3% (11), this rate varies in the literature. In another study on this subject, this rate was reported to be 0.4-33.8% (28). It has also been reported that the gallbladder should be examined completely when dysplasia was observed in the first examination, while there is a study indicating that 4 samples may be sufficient in such cases (25). The authors of this study indicated that the low rate of dysplasia in their series (<0.5%) is due to the lower risk for dysplasia of their patient population than other studies or insufficient sampling / low number of samples (i.e. 1 cassette with 2 samples). In a study of Adsay et al. (12), which was conducted in 2013, it was stated that the risk in the population should be taken into account when looking for a neoplastic lesion as the neoplastic lesions cannot always be detected easily, especially during the macroscopic examination. They also emphasized the important knowledge about the presence of various epithelial lesions in association with neoplastic lesions that can be seen in gallbladder. In the recent consensus meeting held in 2015, it was concluded that at least 3 samples should be obtained from routine cholecystectomy materials including the ductus cysticus margin in the geographic regions where the incidence of gallbladder cancer is high, while materials bearing dysplasia or cancer should be fully examined (29).

There are several studies conducted on the frequency of epithelial anomalies and sampling methods in our country (4,30). Argon et al. (4) recommend that a longitudinal sample starting from the neck of the gallbladder to the fundus should be obtained and placed into the cassette as a rolled-up (Swiss roll) figure. Higher rates of pyloric metaplasia, intestinal metaplasia, low-grade dysplasia, and invasive carcinoma were reported with this method compared to the method where the fundus and the body were examined separately. Bolat et al. (30) reported increased rates of metaplasia, dysplasia, epithelial hyperplasia and inflammation by increasing the number of samples obtained from cholecystectomy material. Several methods were suggested in the literature about how macroscopic sampling should be done and the methods to be followed when epithelial atypia is detected (10,13,31).

In our study, the mean number of blocks was 4 in AS cases. In the study of Wrenn et al. (20), which includes the cost analysis, the rate of important pathological lesions detected by histopathological examination of cholecystectomy materials was found to be low. Nonetheless, it is necessary to continue to perform histopathological examination in the light of the cost analysis. The authors assert that evaluation of risk factors, intra-operative findings and on-table evaluation of the materials may be an alternative approach (20). In an activity-based cost study conducted in Turkey, the laboratory cost of pathology materials was compared to prices indicated in the Healthcare Regulation Report (32). According to this study, cholecystectomy materials have one of the lowest costs among pathology materials. Given the prevalence and low cost in addition to the importance of early detection of malignancies, it can be assumed that AS is not a significant burden for an institution. However, there is no large-scale study on this topic in our country.

The limitations of our study can be listed as the lack of a standard way for creating the database (i.e. mismatched terminology of the lesions among the institutes or lesions found to be too unimportant to mention in the pathology reports), lack of expert consultation by a single qualified expert for dysplastic lesions; and the lack of demographic, socioeconomic, pre- and postoperative follow-up data and cost-effectiveness analysis of AS during the pathological examination of cholecystectomy specimens.

In conclusion, it was found that obtaining new samples from the gallbladder after the first microscopic examination of material contributed to the diagnosis. In addition, the importance of specifying and using standard criteria for macroscopic and microscopic examination was emphasized in the current macroscopy guide of the HPB Pathology Study Group (23). In our study, it was concluded that all pathological findings observed in the cholecystectomy material must be specified in the pathology report.

## CONFLICT of INTEREST

The authors declare no conflict of interest.

## References

[ref-1] Pathologists Royal College of (2005). Histopathology and cytopathology of limited or no clinical value, in Report of Working Group of the Royal College of Pathologists.

[ref-2] Talreja Vikash, Ali Aun, Khawaja Rabel, Rani Kiran, Samnani Sunil Sadruddin, Farid Farah Naz (2016). Surgically Resected Gall Bladder: Is Histopathology Needed for All?. Surg Res Pract.

[ref-3] Sikora Sadiq S., Singh Rajneesh K. (2006). Surgical strategies in patients with gallbladder cancer: nihilism to optimism. J Surg Oncol.

[ref-4] Argon Asuman, Yağcı Ayşe, Taşlı Funda, Kebat Tulu, Deniz Senem, Erkan Nazif, Kitapçıoğlu Gül, Vardar Enver (2013). A different perspective on macroscopic sampling of cholecystectomy specimens. Korean J Pathol.

[ref-5] Albores-Saavedra J, Henson DE, Klimstra DS (2000). Tumors of the gallbladder, extrahepatic bile ducts, and ampulla of Vater. Atlas of Tumor Pathology.

[ref-6] Rosai J (2011). Appendix E: Guidelines for handling of most common and important surgical specimens. Rosai and Ackerman's Surgical Pathology.

[ref-7] Allen Derek C., Cameron R. Iain, Loughrey Maurice B., Allen Derek C., Cameron R. Iain (2013). Gallbladder. Histopathology Specimens.

[ref-8] Abraham S, Westra WH, Hruban RH, Phelps TH, Isacson C (2002). Gallbladder and extrahepatic biliary system. Surgical Pathology Dissection: An Illustrated Guide.

[ref-9] Esendağlı Güldal, Akarca F. Göknur, Balcı Serdar, Argon Asuman, Erhan Selma Şengiz, Turhan Nesrin, Zengin Neslihan İnce, Keser Sevinç Hallaç, Çelik Betül, Bulut Tangül, Abdullazade Samir, Erden Esra, Savaş Berna, Bostan Temmuz, Sağol Özgül, Ağalar Anıl Aysal, Kepil Nuray, Karslıoğlu Yıldırım, Günal Armağan, Markoç Fatma, Saka Burcu, Özgün Gonca, Özdamar Şükrü Oğuz, Bahadır Burak, Kaymaz Esin, Işık Emre, Ayhan Semin, Tunçel Deniz, Yılmaz Banu Özgüven, Çelik Sevinç, Karabacak Tuba, Seven İpek Erbarut, Çelikel Çiğdem Ataizi, Gücin Zuhal, Ekinci Özgür, Akyol Gülen (2018). A Retrospective Evaluation of the Epithelial Changes/Lesions and Neoplasms of the Gallbladder in Turkey and a Review of the Existing Sampling Methods: A Multicentre Study. Turk Patoloji Derg.

[ref-10] Adsay NV, Mills SE (2015). Gallbladder, extrahepatic biliary tree, and ampulla. Sternberg's Diagnostic Surgical Pathology.

[ref-11] Albores-Saavedra Jorge, Henson Donald Earl, Klimstra David S. (2015). Benign epithelial tumors of the gallbladder. Tumors of the gallbladder, extrahepatic bile ducts, and vaterian system.

[ref-12] Adsay Volkan, Saka Burcu, Basturk Olca, Roa Juan Carlos (2013). Criteria for pathologic sampling of gallbladder specimens. Am J Clin Pathol.

[ref-13] Adsay NV, Klimstra DS, Odze RD, Goldblum JR (2015). Benign and malignant tumors of the gallbladder and extrahepatic biliary tract. Odze and Goldblum Surgical Pathology of the GI Tract, Liver, Biliary Tract, and Pancreas.

[ref-14] Dursun N, Saka B, Balci S, Bagci P, Roa JC, Araja JC, Basturk O, Terry P, Minhas F, Ducato L, Adsay V (2014). Biologic behavior of gallbladder high-grade dysplasia: A long-term survival analysis of 125 cases elucidates a mostly curable disease, which is marker of biliary tract cancer risk in some patients. Mod Pathol.

[ref-15] Patel K, Balci S, Saka B, Knight J, Basturk O, Sarmiento J, Roa JC, Araya JC, Sweeney J, Terry P, Goodman M, Adsay V (2014). "Carcinoma In-Situ" of the gallbladder: The SEER Database Perspective. Mod Pathol.

[ref-16] Lee Seung Eun, Jang Jin-Young, Lim Chang-Sup, Kang Mee Joo, Kim Sun-Whe (2011). Systematic review on the surgical treatment for T1 gallbladder cancer. World J Gastroenterol.

[ref-17] Reid Kaye M., Medina Antonio, Donohue John H. (2007). Diagnosis and surgical management of gallbladder cancer: a review. J Gastrointest Surg.

[ref-18] Fong Y., Heffernan N., Blumgart L. H. (1998). Gallbladder carcinoma discovered during laparoscopic cholecystectomy: aggressive reresection is beneficial. Cancer.

[ref-19] Foster Jason M., Hoshi Hisakazu, Gibbs John F., Iyer Renuka, Javle Miland, Chu Quyen, Kuvshinoff Boris (2007). Gallbladder cancer: Defining the indications for primary radical resection and radical re-resection. Ann Surg Oncol.

[ref-20] Wrenn Sean M., Callas Peter W., Abu-Jaish Wasef (2017). Histopathological examination of specimen following cholecystectomy: Are we accepting resect and discard?. Surg Endosc.

[ref-21] Lam C. M., Yuen A. W., Wai A. C., Leung R. M., Lee A. Y., Ng K. K., Fan S. T. (2005). Gallbladder cancer presenting with acute cholecystitis: a population-based study. Surg Endosc.

[ref-22] Sun Chuan Dong, Zhang Bing Yuan, Wu Li Qun, Lee Woo Jung (2005). Laparoscopic cholecystectomy for treatment of unexpected early-stage gallbladder cancer. J Surg Oncol.

[ref-23] (2017). Patoloji Dernekleri Federasyonu Hepato-Pankreato-Biliyer Patoloji Çalışma Grubu Makroskopi Kılavuzu 2017 Güncellemesi.

[ref-24] (2020). Protocol for the examination of specimens from patients with carcinoma of the gallbladder. Version 4.1.0.0.

[ref-25] Renshaw Andrew A., Gould Edwin W. (2012). Submitting the entire gallbladder in cases of dysplasia is not justified. Am J Clin Pathol.

[ref-26] Akki Ashwin S., Zhang Wei, Tanaka Kathryn E., Chung Sun M., Liu Qiang, Panarelli Nicole C. (2019). Systematic Selective Sampling of Cholecystectomy Specimens Is Adequate to Detect Incidental Gallbladder Adenocarcinoma. Am J Surg Pathol.

[ref-27] Adsay V, Roa JC, Basturk O, Torres J, Mucientes F, Del Pozo M, Villaseca MA, Aguayo G, Bellolio ER, Araya JC, Endo I, Lee K, Jang KT, Jang JY, Ohike N, Shimizu M, Hirabayashi K, Terris B, Zamboni G, Reid M, Xue Y, Bedolla G, Quigley B, Krasinskas A, Akkas G, Memis B, Klimstra D, Hruban RH, Zhu B, Van Dyke AL, Koshiol J (2016). Epithelial atypia in the gallbladder: Diagnosis and classification in an international consensus study. Mod Pathol.

[ref-28] Sasatomi E., Tokunaga O., Miyazaki K. (2000). Precancerous conditions of gallbladder carcinoma: overview of histopathologic characteristics and molecular genetic findings. J Hepatobiliary Pancreat Surg.

[ref-29] Aloia Thomas A., Járufe Nicolas, Javle Milind, Maithel Shishir K., Roa Juan C., Adsay Volkan, Coimbra Felipe J. F., Jarnagin William R. (2015). Gallbladder cancer: expert consensus statement. HPB (Oxford).

[ref-30] Bolat F, Kayaselcuk F, Nursal TZ, Bal N, Tuncer I (2007). The correlation of the histopathological findings by increasing the sample size in cholecystectomies. Turk Patoloji Derg.

[ref-31] Hartman Douglas, Krasinskas Alyssa M., Sasatomi Eizaburo (2013). Caveat emptor: submitting the entire gallbladder in cases of dysplasia is not justified. Am J Clin Pathol.

[ref-32] Ergün Ferda A. K., Ağirbaş Ismail, Kuzu Işınsu (2013). Activity-based costing for pathology examinations and comparison with the current pricing system in Turkey. Turk Patoloji Derg.

